# Why Do Applicants (Dis)Like Selection Methods? The Role of Stimulus and Response Format for Need Satisfaction

**DOI:** 10.1007/s10869-024-10002-7

**Published:** 2025-01-23

**Authors:** Valerie S. Schröder, Martin Kleinmann, Anna Luca Heimann, Pia V. Ingold

**Affiliations:** 1 Department of Psychology, University of Zurich, Binzmühlestrasse 14/12, 8050 Zurich, Switzerland; 2 Department of Psychology, University of Copenhagen, Copenhagen, Denmark

**Keywords:** Personnel selection, Applicant reactions, Self-determination theory, Content-method confusion, Predictor method factors

## Abstract

In today’s employment market, it is important to use selection instruments that resonate positively with applicants. To advance the theoretical understanding of why applicants react differently to different selection instruments, this study examines how the satisfaction of basic psychological needs influences applicant reactions, specifically perceived interpersonal warmth and opportunity to perform. Using a 2 × 2 between-subjects design, this study systematically manipulated two method factors, namely, the stimulus format (written vs. interactive) and the response format (open-ended vs. close-ended) of selection instruments that were designed to measure the same personality traits. Drawing on self-determination theory, this study proposes and tests the satisfaction of the needs for relatedness, autonomy, and competence as a mechanism to explain the relationships between specific method factors and applicant reactions. Regarding the stimulus format, results indicated no effect on need satisfaction, but a positive effect of an interactive stimulus format on interpersonal warmth, when combined with an open-ended response format. Regarding the response format, results indicated that an open-ended response format increased perceptions of opportunity to perform, mediated by greater satisfaction of the need for autonomy. Practical implications for the design of selection instruments are presented.

To attract top talents, it is crucial for organizations to use selection instruments that applicants perceive positively (Chapman et al., 2005; McCarthy et al., 2017). A large body of research compares applicant reactions to different selection instruments; for example, research suggests that organizations are likely to elicit more positive applicant reactions when they use job interviews and more negative applicant reactions when they use personality inventories as selection instruments (e.g., Anderson et al., 2010; Hausknecht et al., 2004; Ni & Hauenstein, 1998). However, little is known about (a) which individual methodological features of a selection instrument influence applicant reactions and (b) the psychological process that explains why applicants react the way they do. This lack of knowledge hinders more specific advice on the design of selection instruments, limiting the impact of applicant reaction research (Ryan & Huth, 2008).

The present study aims to create a better understanding of applicant reactions by examining how different methodological features of selection instruments influence need satisfaction as mediators for applicant reactions. Research has only recently started to look at psychological processes as explaining mechanisms for applicant reactions (Borman et al., 2023; Buil et al., 2020; Dalal et al., 2021) and specifically the satisfaction of basic psychological needs as defined by self-determination theory (SDT; Deci & Ryan, 2000, 2010). According to SDT, everyone is endowed with three basic needs: need for relatedness, need for competence, and need for autonomy. The satisfaction of these basic needs determines the motivation to complete a given selection instrument (Buil et al., 2020). Beyond the known, exploring the role of individual features of selection instruments (i.e., method factors) for satisfaction of specific needs can help to understand why applicants prefer certain selection instruments over others. For example, applicants may react negatively to a selection instrument because it allows for too little autonomy or feeling of competence due to its restricted response format, as has been shown for forced-choice personality inventories (Borman et al., 2023), decreasing their perception that the instrument is a useful and necessary part of a selection process.

The present research uses an experiment design to generate insights into what causes applicant reactions and need satisfaction of specific selection instruments by attributing them to two method factors: the stimulus format (i.e., how the content of a selection instrument is presented to applicants, in a written vs. interactive format) and the response format (i.e., how applicants can respond to the selection instrument, in an open-ended vs. close-ended format). Going beyond previous research (Borman et al., 2023; Buil et al., 2020), this study proposes different mediators for different applicant reaction dimensions. We propose that need for relatedness mediates the relationship between stimulus format and interpersonal warmth, and that need for autonomy and need for competence mediate the relationship between response format and opportunity to perform. In the past, critiques have alluded that “[w]ithout holding all else constant save content and then exploring systematic variations in how that content is assessed, it is hard to determine what applicants are reacting to” (Ryan & Huth, 2008, p. 120). Using a method factor approach, that is, extracting the effects of different methodological features (Lievens & Sackett, 2017) of otherwise identical instruments, this study will answer this call. As content, we will assess personality traits because they are commonly assessed selection predictors (Rothstein & Goffin, 2006), but traditional personality measures are often perceived negatively by applicants (Anderson et al., 2010; Ni & Hauenstein, 1998). Keeping the personality construct constant and adjusting methodological features will help to identify levers to improve negative reactions to personality measures.

Combining SDT with a method factor approach, this study sets out to enhance our theoretical understanding on the processes underlying positive applicant reactions. This may allow for designing or adapting selection instruments for greater need satisfaction and more positive applicant reactions. The applied method factor approach will contribute to a more fine-grained understanding of applicant reactions. For example, it will clarify whether the interactive stimulus format and the open-ended response format that are typical of job interviews contribute to their favorability by fulfilling specific needs. Applying SDT to the selection context will shed light on the psychological processes underlying applicant reactions. This is because it allows us to provide knowledge on the role of satisfaction of specific psychological needs (i.e., needs for relatedness, autonomy, and competence) as mediators between method factors and different types of applicant reactions (i.e., interpersonal warmth and opportunity to perform). Understanding the mechanisms underlying applicant reactions and their triggers will ultimately allow for theory development in the area of applicant reactions and more theoretically driven decisions in the design and choice of selection instruments.

## Applicant Reactions to Different Selection Instruments

Although the main goal of a selection instrument is to make a valid prediction of an applicant’s future job performance, ensuring that a given instrument elicits positive applicant reactions can prevent organizations from negative consequences such as qualified applicants losing interest in the job because they had negative experiences during the selection process (McCarthy et al., 2017). Indeed, research has shown that how applicants perceive the selection process corresponds to their perceptions of the attractiveness of the organization (Chapman et al., 2005; Hausknecht et al., 2004; Uggerslev et al., 2012) and their intentions to accept a job offer (Chapman et al., 2005; Hausknecht et al., 2004).

Applicant reactions can be conceptualized along different dimensions (Gilliland, 1993; Steiner & Gilliland, 1996). Among the commonly distinguished applicant reaction dimensions, some relate more to the content of a selection instrument (e.g., perceptions of the scientific validity of the method or employers’ right to obtain information) or to context factors (e.g., perceptions on common use of the instrument or privacy concerns). The two dimensions that appear particularly relevant for the methodological features of a selection instrument are *interpersonal warmth*, which describes how personal a given instrument is, for example, because it allows for interpersonal interaction, and *opportunity to perform*, which describes the extent to which a selection instrument allows applicants to demonstrate their strengths (Gilliland, 1993). Both reaction dimensions have demonstrated influence on relevant outcomes such as the intentions to accept a job offer, to recommend the organization to others as an employer (Bauer et al., 1998; Georgiou, 2021; Hausknecht et al., 2004), or the chances of an applicant to reapply (LaHuis et al., 2007), strengthening their relevance in the selection process.

Applicant reaction research has repeatedly shown that applicants react more positively to certain instruments as compared to others concerning opportunity to perform and interpersonal warmth (Anderson et al., 2010; Gilliland & Steiner, 2012; Hausknecht, 2013; Hausknecht et al., 2004; Hoang et al., 2012; Moscoso & Salgado, 2004; Nikolaou & Judge, 2007; Steiner & Gilliland, 1996). Generally, a consistent pattern shows that interviews and work samples rank among the most favorable selection instruments in general, and specifically on interpersonal warmth and opportunity to perform (Anderson et al., 2010; Hoang et al., 2012). In contrast, instruments that lack interaction, such as cognitive tests or integrity tests, are perceived as impersonal or cold and as providing less opportunity to perform. Yet, the specific reasons for the differences in these applicant reactions, that is, why applicants react differently and what elicits this reaction, remain unknown.

### Studying Applicant Reactions to Specific Method Factors

To answer the question what applicants react to, the modular framework on method factors by Lievens and Sackett (2017) offers a useful lens. In their framework, they propose that certain features of a selection instrument might be decisive for selection outcomes such as applicant reactions. Therefore, they systematically decompose selection methods into method factors to provide an “improved insight into the isolated workings of the different components underlying selection procedures” (p.1). For example, without being further specified, common findings on applicant reactions can inform which selection instruments are most favorable, yet they leave open what the key factors are that make one selection instrument (e.g., an interview) a more favorable experience for applicants, compared to other selection instruments (e.g., personality inventories). Because empirical work has demonstrated that adjusting method factors can be a tool to enhance applicant reactions (Bowen et al., 2002; Converse et al., 2008; Lievens et al., 2015), identifying the method factors that make interviews especially favorable can be useful in guiding the redesign of less favorable selection instruments by making only a few format-related changes.

Personality measurement in particular offers the chance for enhanced applicant reactions by adjusting method factors. Personality traits are commonly used predictor constructs (Rothstein & Goffin, 2006) yet have a reputation for provoking negative reactions from applicants (Anderson et al., 2004; Ni & Hauenstein, 1998). Personality inventories are rather impersonal and leave little opportunity to express one’s eligibility for a job (Lievens, 2017). Adjusting unfavorable method factors in personality inventories can be a valuable approach to enhance the favorability of their measurement. Measuring personality through alternative methods, such as Situational Judgment Tests (Mussel et al., 2018; Olaru et al., 2019; Oostrom et al., 2019) or interviews (Heimann et al., 2021a, 2021b; Van Iddekinge et al., 2005), has produced promising results, and adjusting the method factors characteristic to these alternative methods that drive positive applicant reactions offers great potential to efficiently enhance the favorability of its assessment.

Two method factors in specific may be useful to enhance the perceived opportunity to perform and interpersonal warmth of personality measurement, namely (a) the *stimulus format* and (b) the *response format.* Stimulus format and response format are relevant method factors that characterize common selection instruments (e.g., interviews and personality inventories; Armoneit et al., 2020). The *stimulus format* describes how a test item (e.g., an interview question, a personality item, a cognitive task) is presented to applicants. There is no general categorization for stimulus formats across selection instruments, but there are specific categorizations for individual selection instruments (Lievens & Sackett, 2017). For example, personality inventories are typically presented in a textual stimulus format, and interviews are typically presented by an interviewer in an interactive stimulus format. As common stimulus formats across selection instruments, Lievens and Sackett (2017) list textual, pictorial, auditory, dynamic audiovisual, and interactive stimuli, with the latter being either face-to-face interactive or remote interactive (i.e., as a videoconference). The stimulus format plays an important role in the information that a selection instrument conveys, that is, if it transports only question content, or additional information, such as audio or visual information (Daft & Lengel, 1986). For this study, we will compare a *textual* stimulus format (i.e., common for personality inventories) with an *interactive* stimulus format (i.e., a videoconference or remote interactive format, common for interviews) as they represent opposite ends of the media richness continuum. The *response format* describes how applicants respond to the question. In this study, we will compare whether applicants choose a predefined response (close-ended response format, common for personality inventories) or freely formulate their own response (open-ended response format, common for interviews), which corresponds to the established way to categorize response formats (Lievens & Sackett, 2017).

We hypothesize that an interactive stimulus format will elicit more positive perceptions of interpersonal warmth because it allows for personal contact with another person. The stimulus format determines how many social cues a selection instrument transports (Potosky, 2008) and might therefore play a more pivotal role to applicant reactions in terms of how personal a selection instrument is perceived (i.e., interpersonal warmth), as compared to how they can perform in it (opportunity to perform). In job interviews, the stimulus format allows the applicant to see and hear the person who presents the interview questions. Although the medium by which the stimulus is transported is now more often technology-mediated (Blacksmith et al., 2016; Chapman et al., 2003), the interactive stimulus format remains a key feature of the interview (with few exceptions, e.g., some asynchronous video interviews present the question via text; Basch et al., 2022; Brenner et al., 2016). Because the applicant can see and hear the interviewer, the videoconference presentation creates a social situation that allows them to get to know someone from the organization.

- *Hypothesis 1*: Perceptions of interpersonal warmth will be more positive in an interactive than in a written stimulus format.

We hypothesize that an open-ended response format will elicit more positive perceptions of opportunity to perform because it gives the applicant full control over the information they provide. By determining the cues that applicants can send, response format might play a more pivotal role for applicant reactions in terms of opportunity to perform compared to interpersonal warmth. If the response format is open-ended, this will likely increase perceptions of opportunity to perform because by formulating their own response, applicants can decide what information they share with the interviewer, giving them the freedom to emphasize the competencies and skills they want to demonstrate.

- *Hypothesis 2*: Perceptions of opportunity to perform will be more positive in an open-ended than in a close-ended response format.

### Differences in Need Satisfaction Depending on Method Factors

To answer the question why applicants react to selection instruments in a given way, SDT (Deci & Ryan, 2000; Deci et al., 2001, 2017) offers potential explaining mechanisms. The SDT is a motivational framework that describes drivers of human motivation and well-being. According to this theory, an individual can be *autonomously motivated* to engage in an activity, that is, they volitionally engage in this activity out of their own conviction. A state of autonomous motivation is achieved when three needs are satisfied: *need for relatedness* describes the need to feel connected to others. *Need for autonomy* describes the need to have a free choice in one’s own actions. *Need for competence* describes the need to experience one’s own doing as being effective and mastering tasks. These three needs are fundamental to SDT because they are the essential mediators between an activity or environment an individual is confronted with and their resulting motivation. Highlighting the relevance of basic needs in the context of work, research has shown that their satisfaction influences affective experiences such as stress and well-being (Andreassen et al., 2010; Deci et al., 2001, 2017; Gillet et al., 2012; Greguras & Diefendorff, 2009; Olafsen et al., 2017; Vansteenkiste et al., 2007) and also job performance (Baard et al., 2004; Deci et al., 2017).

Applying SDT to the selection context, different applicant reactions to different selection instruments can be explained by the degree to which completing a given selection instrument allows for the satisfaction of the applicant’s basic needs. In selection, applicants’ primary motivation to complete a given selection instrument is extrinsic, meaning that the completion is a necessary mean for getting a desired job. If a selection instrument satisfies applicants’ basic needs, they will be autonomously motivated for its completion, that is, they will complete it out of a conviction that the instrument is useful and important for the selection process (Buil et al., 2020). For example, the need for relatedness may be satisfied if a selection instrument allows for connecting with other people such as during an honest conversation as part of a selection process. The need for autonomy may be satisfied if a selection instrument gives the applicant free choice in how they want to portray themselves such as responding to open-ended questions about the applicant’s personality. The need for competence may be satisfied if the applicant has the chance to demonstrate how well they can master difficult situations such as when responding to questions about challenges in their past. In sum, the satisfaction of basic needs when completing a selection instrument is thought to contribute to a positive experience for applicants. Accordingly, they are likely to react more positively to the instrument.

First, empirical work has considered need satisfaction as an antecedent of applicants’ favorability perceptions of selection instruments (Borman et al., 2023; Buil et al., 2020). As a first empirical study, Buil et al. (2020) demonstrated the role of applicants’ need satisfaction for the acceptance of gamified selection instruments. They found that the satisfaction of need for autonomy and competence affected the perceived ease of use of a business simulator, which led to a better attitude towards the instruments and higher satisfaction, intentions to recommend the organization, and organizational attractiveness. Borman et al. (2023) compared how need satisfaction changed when participants were allowed to comment on or review their responses after a forced-choice personality test. They found that being able to review and adjust responses enhanced the satisfaction of the needs for autonomy and competence. Yet, even in the variation of the personality inventory where participants were allowed to adjust and review their response to the forced-choice response format, participants’ need satisfaction was still lower compared to their need satisfaction when completing a selection instrument with a traditional Likert-scale response format. Overall, these findings demonstrate the explanatory role of need satisfaction in applicant reactions to selection instruments.

This study extends the use of SDT in the selection context by combining it with a method factor approach, and will elucidate to what degree method factors of selection instruments are responsible for satisfying different applicant needs. Buil et al. (2020) and Borman et al. (2023) started exploring the psychological process underlying applicant reactions using SDT. While these studies have provided relevant knowledge on how fair applicants perceive an instrument in general, the current study provides knowledge on the specific connections between certain needs and specific applicant reaction dimensions. Specifically, this study maps the satisfaction of specific needs to specific applicant reactions (i.e., the need for relatedness to interpersonal warmth and the needs for autonomy and competence to opportunity to perform). Furthermore, it elucidates the role of method factors for need satisfaction and applicant reactions. Going beyond Borman et al. (2023), it systematically varies two method factors that are characteristic to many selection instruments. Thus, this study will provide fine-grained knowledge on the factors and processes leading to need satisfaction in personnel selection.

Specifically, this study investigates how the satisfaction of the needs (for relatedness, autonomy, and competence) explains the effects of method factors (stimulus and response formats), on applicant reactions (perceived opportunity to perform and interpersonal warmth). First, we expect that an interactive stimulus format will better satisfy applicants’ need for relatedness as compared to a written stimulus. For one, an interactive stimulus format implies the presence of another person. Further, the stimulus format can affect the amount of social cues being sent to this person (Potosky, 2008). Therefore, it is primarily relevant for the need for relatedness, as opposed to needs that are more focused on one’s own doing (i.e., need for autonomy and competence). Based on SDT, the need for relatedness is satisfied when individuals have the impression that they can build a relationship with another person (Deci & Ryan, 2000). Selection instruments with an interactive stimulus during which applicants share the assessment experience with, for example, an interviewer, may lead to an experience that feels more social and reciprocal (Celani & Singh, 2011). Reversely, reduced opportunities for interaction with an interview partner may hurt the satisfaction of need for relatedness.

- *Hypothesis 3*: An interactive stimulus format will better satisfy need for relatedness compared to a written stimulus format.

Second, we suggest that an open-ended response format better satisfies applicants’ needs for autonomy and competence. An open-ended response implies that an applicant can choose how to respond to a stimulus, that is, the content and how they phrase it. In contrast, in the close-ended response format, they chose from response options that are predefined. Because the response format determines the cues that the applicant can send, it is supposed to be more relevant for applicants’ needs related to their actions and performance, and potentially less relevant for need for relatedness. According to SDT, the need for autonomy is satisfied when one is free in their choices such that it is in accordance with their self-understanding (Buil et al., 2020; DeCharms, 1968; Deci et al., 2017). The more choices a person has, the more autonomy they experience (Zuckerman et al., 1978). Therefore, an open-ended response format allows applicants to sketch a response that better aligns with their self-understanding regarding preferences and behaviors and to add explanations or examples, which may lead to experiencing more autonomy.

Based on SDT, the need for competence is satisfied when one overcomes challenges and feels personally responsible for their performance (Deci & Ryan, 2000; Fisher, 1978). The open-ended response format may be perceived as more satisfying in this regard because it allows applicants to better emphasize the competencies they possess. The open-ended response format allows applicants to emphasize their favorable personal characteristics and skills and how they come into play in the context of work. In contrast, in the close-ended response format, applicants may feel that they are prevented from mentioning their individual competencies, resulting in a lower satisfaction of this need.

- *Hypotheses 4a and b*: An open-ended response format will better satisfy need for (a) autonomy and (b) competence as compared to a close-ended response format.

A higher satisfaction of need for relatedness may explain why applicants perceive more interpersonal warmth in instruments with an interactive stimulus format. Based on SDT (Deci & Ryan, 2000, 2010), need for relatedness is satisfied when there is a perceived warmth in a situation (Vansteenkiste et al., 2020). Accordingly, the degree to which the method factors characteristic to an instrument can satisfy the need for relatedness may differentiate instruments that elicit high or low perceptions of interpersonal warmth*.* Relatedly, empirical evidence from applicant reaction research suggests that the degree to which instruments incorporate the appearance of another person makes a difference, as applicant reactions tend to be more favorable for interviews and less favorable for cognitive ability tests or personality inventories (Anderson et al., 2010). To date, it has never been empirically tested (a) whether an interactive stimulus format satisfies the need for relatedness (see our Hypothesis 3) and (b) whether the satisfaction of the need for relatedness leads applicants to perceive more interpersonal warmth (see our Hypothesis 5). Yet, empirical research outside the context of selection and assessment has shown that the satisfaction of the need for relatedness increases a sense of community and decreases loneliness (Lin, 2016). Combined with our previous reasoning, we conclude that applicants' perceptions of interpersonal warmth in methods with an interactive versus written stimulus format are mediated by the satisfaction of need for relatedness.

- *Hypothesis 5*: Satisfaction of need for relatedness will mediate the relationship between an interactive stimulus format and perceptions of interpersonal warmth.

Analogously, the satisfaction of needs for autonomy and competence may explain the relationship between an open-ended response format and perceived opportunity to perform. Based on SDT, the degree to which the method factors of an instrument satisfy need for autonomy and competence may differentiate instruments that elicit high or low perceptions of opportunity to perform. Specifically, SDT suggests that frustration of the need for autonomy can lead to feelings of being controlled (Deci & Ryan, 1987) and “feeling pushed in an unwanted direction” (Vansteenkiste et al., 2020, p. 3), and that frustration of the need for competence can lead to feelings of ineffectiveness, both of which counteract a perceived opportunity to perform. In other words, instruments that provide less opportunity to perform may be characterized by method factors that frustrate autonomy or competence needs. Empirical evidence comparing different selection instruments suggests that the degree of freedom to which applicants can decide how to complete a task and what information to share could make a difference, as applicants tend to perceive a greater opportunity to perform in interviews or work samples and less opportunity to perform in instruments with more rigid test formats (Anderson et al., 2010). Yet, this is only indirect evidence given that this has not been tested for method factors. Explicitly, it has never been empirically tested (a) whether an open-ended response format satisfies the needs for autonomy and competence (see Hypotheses 4a and b) and (b) whether the satisfaction of the needs for autonomy and competence leads applicants to perceive a greater opportunity to perform in a selection instrument (see Hypotheses 6a and b). Empirical research outside the context of selection and assessment suggests that higher satisfaction of the needs for autonomy and competence relates to higher performance (Van den Broeck et al., 2016) and this suggests that need satisfaction may also be relevant to a perceived opportunity to perform. Therefore, we propose a mediation mechanism in which opportunity to perform perceptions are higher in methods with open-ended as opposed to closed-ended response formats because these response formats satisfy the needs for autonomy and competence:

- *Hypothesis 6a and b*: Need for (a) autonomy and (b) competence will mediate the relationship between an open-ended response format and perceptions of opportunity to perform.

## Methods

### Sample

A total of 208 participants (64% female) took part in a simulated selection procedure that was conducted online via a videoconference system. The study was advertised as a job application training (similar to previous studies, e.g., Ingold et al., 2015; Roulin & Powell, 2018; Swider et al., 2016). The job application program consisted of a short, simulated selection procedure conducted remotely via Zoom. Participants indicated that they were seeking job application training either after completing their education (64.94%) or due to a job change (13.46%). Participants were recruited through two main channels: universities and social media. For university recruitment, we contacted the career services and administrative staff of various departments at several German-speaking universities (i.e., universities in Switzerland, Germany, and Austria) and asked them to forward information about the job application training we offered to anyone who might be interested (e.g., students or alumni). To this end, we provided the career services and administrative staff with advertising material (i.e., a flyer and a draft for a recruitment email). In most cases, our advertising materials were then distributed to students and alumni via mailing lists. The emails included information about our website, where participants could sign up directly for the study. For social media recruitment, we posted information about the job application program on professional (LinkedIn and Xing) and non-professional (Instagram) platforms to reach a wider audience. The majority of participants (87.02%) reported that they had heard about the training through their university (e.g., through a newsletter).

The mean age of participants was 28.04 (*SD* = 5.91) years. The majority of participants (72.12%) held a university degree (Bachelor’s degree or higher), and they had on average 4.73 (*SD* = 5.24) years of job experience. Concerning experience with videoconference systems, participants indicated that they used such systems on average weekly (*M* = 2.89, *SD* = 1.22 with 2 indicating monthly and 3 indicating weekly) and felt confident about using them (*M* = 5.08, *SD* = 1.50, rated on a scale from 1–*not at all confident* to 7–*very confident*).

### Procedure

We used a 2 × 2 between-subjects design in which participants were randomly assigned to one of four conditions, with either interactive or written stimulus format and either open-ended or close-ended response format. All conditions started with an introduction by two test directors. For the interactive conditions, participants remained in the videoconference where the questions were presented by test directors. In the written conditions, participants proceeded to an online questionnaire tool. After completing the instrument, participants rated their need satisfaction and applicant reactions.

The procedure was conducted as a simulated selection procedure, which is a common paradigm in the context of personnel selection research (e.g., Bourdage et al., 2020; Heimann & Schmitz-Wilhelmy, 2024; Ingold et al., 2015; Jansen et al., 2013; Klehe et al., 2008; Roulin & Powell, 2018; Swider et al., 2016) in which participants are instructed and incentivized to behave like applicants, while the setting still allows to experimentally manipulate various selection-related factors. To create a realistic setting, participants were asked to dress as they would for a real job interview. Further, there was a job profile sent to interviewees before their interview, to provide a common frame-of-reference and to allow interviewees to prepare themselves. The job profile is displayed in Fig. 6 in the Appendix. Participants indicated that they perceived the simulated selection setting as realistic (*M* = 5.32, *SD* = 1.41), could adapt to the role as an applicant (*M* = 5.74, *SD* = 1.16), felt as if they were a real applicant (*M* = 5.41, *SD* = 1.31), and behaved as they would in a real selection setting (*M* = 6.04, *SD* = 0.95 with all responses on a scale from 1—*completely disagree* to 7—*completely agree*).

### Experimental Manipulation

Conditions were systematic combinations of stimulus format (interactive vs. written) and response format (close-ended vs. open-ended response format) resulting in four conditions: a written /close-ended condition (Group 1), an interactive/close-ended condition (Group 2), a written/open-ended condition (Group 3), and an interactive/open-ended condition (Group 4). Thus, the written/close-ended condition was similar to a traditional situational judgment test, the interactive/close-ended condition can be viewed as a situational judgment test that was read out loud via videoconference to the participants, the written/open-ended condition was similar to a written situational interview, and the interactive/open-ended condition was similar to a situational job interview via videoconference. The different conditions are displayed in Table 1. There were 53 participants in Group 1, 54 participants in Group 2, 51 participants in Group 3, and 50 participants in Group 4.

**Table 1:** Study design with four experimental conditions

		Stimulus format	
		Written	Interactive
Response format	Close-ended	Condition 1 Written presentation of hypothetical job situations on an online platform. Participants choose one of five response options describing their potential behavior in this situation (similar to a written situational judgment test)	Condition 2 Hypothetical job situations read aloud by the test leader. Participants choose one of five response options describing their potential behavior in this situation (similar to a videoconference situational judgment test)
	Open-ended	Condition 3 Written presentation of hypothetical job situations on an online platform. Participants openly describe how they would react in this situation (similar to a written situational interview)	Condition 4 Hypothetical job situations read aloud by the test leader. Participants openly describe how they would react in this situation (similar to a videoconference situational interview)

The basis for all conditions was the same situational questions measuring the same personality traits (displayed in the appendix in Table 6). We chose personality as a construct because it is a popular selection predictor (Rothstein & Goffin, 2006) that can be measured with different selection methods or variations thereof (Heimann et al., 2020, 2021a, 2021b; Kasten et al., 2020; Mussel et al., 2018; Olaru et al., 2019; Oostrom et al., 2019). Specifically, each condition presented the participants with identical hypothetical job situations followed by the instruction “How would you feel in this situation and how would you behave?”. Depending on the condition, participants then chose one of five response options each displaying a potential behavior in this situation (close-ended response format) or formulated their own response (open-ended response format).

### Measures

All items for applicant reactions (i.e., interpersonal warmth and opportunity to perform) and need satisfaction were rated on a scale from 1—*completely disagree* to 7—*completely agree*. We ran a confirmatory factor analysis to ensure the distinction of the constructs measured and compared it to three other models in which applicant reactions loaded on the same factors as need satisfaction. The intended model, specifying two applicant reaction dimensions and three need constructs, showed a good fit to the data (*χ*^2^(67) = 117.43, *p* < 0.001; *CFI* = 0.97; *RMSEA* = 0.06, *SRMR* = 0.05) and fitted better compared to the alternative models (compared using AIC because models are not nested).

#### Need Satisfaction

Need satisfaction was measured with three items for each need. All items for need satisfaction are displayed in the appendix in Table 7. Items were adjusted from Buil et al. (2020) so that they were suitable for the experimental manipulation. For example, the item “The method provides me with different options.” for need for autonomy was adapted to “I have full freedom in how I complete this selection instrument.” Cronbach’s alpha was 0.86 for need for relatedness, 0.81 for need for autonomy, and 0.85 for need for competence.

#### Applicant Reactions

Interpersonal warmth was measured with two items: one from Steiner and Gilliland (1996) “The selection instrument is impersonal and cold” and a self-developed item “This instrument creates a personal atmosphere.” The correlation between the two items was 0.70.

Opportunity to perform was measured using the four items from the selection procedural justice scale by Bauer et al. (2001): “I could really show my skills and abilities through this instrument,” “This test allowed me to show what my job skills are,” “This test gives applicants the opportunity to show what they can really do,” and “I was able to show what I can do on this test.” Cronbach’s alpha was 0.88.

## Results

Intercorrelations, means, and standard deviations of all study variables for Conditions 1 and 2 are presented in Table 2 and for Conditions 3 and 4 in Table 3. Analyses were conducted using R and MPlus. Prior to hypothesis testing, we conducted preliminary analyses to ensure that the groups were comparable. We did not find significant differences between groups with respect to gender (*χ*^2^(3) = 1.77, *p* = 0.620), age (*F*(3) = 2.53, *p* = 0.057), experience with videoconference systems (*F*(3) = 0.65, *p* = 0.585), or work experience (*F*(3) = 2.53, *p* = 0.062).

**Table 2:** Correlation and reliability of study variables for Condition 1 written/close-ended and Condition 2 interactive/close-ended

	*M*	*SD*	1	2	3	4	5	6	7
*M*			1.65	28.67	3.41	5.32	2.36	3.19	2.78
*SD*			0.48	6.00	1.39	1.28	1.17	1.47	1.27
1. Gender is female	1.57	0.50		− 0.02	− 0.11	− 0.16	− 0.04	− 0.01	− 0.15
				[− 0.29, 0.25]	[− 0.36, 0.17]	[− 0.41, 0.11]	[− 0.30, 0.23]	[− 0.28, 0.26]	[− 0.40, 0.13]
2. Age	27.25	4.75	0.06		0.05	0.25	− 0.01	0.10	0.06
			[− 0.21, 0.33]		[− 0.22, 0.32]	[− 0.02, 0.48]	[− 0.28, 0.26]	[− 0.17, 0.36]	[− 0.22, 0.32]
3. Need for autonomy	3.81	1.56	0.04	0.06		0.49^**^	0.51^**^	0.52^**^	0.44^**^
			[− 0.23, 0.31]	[− 0.21, 0.33]		[0.26, 0.67]	[0.28, 0.68]	[0.30, 0.69]	[0.20, 0.64]
4. Need for competence	5.58	1.12	0.19	− 0.08	0.33*		0.29*	0.38^**^	0.46^**^
			[− 0.08, 0.44]	[− 0.34, 0.20]	[0.06, 0.55]		[0.03, 0.52]	[0.13, 0.59]	[0.22, 0.65]
5. Need for relatedness	2.62	1.11	− 0.05	0.18	0.53^**^	0.37^**^		0.79^**^	0.42^**^
			[− 0.32, 0.22]	[− 0.10, 0.43]	[0.31, 0.70]	[0.11, 0.58]		[0.67, 0.87]	[0.17, 0.62]
6. Interpersonal warmth	3.70	1.43	0.15	0.19	0.50^**^	0.44^**^	0.55^**^		0.42^**^
			[− 0.13, 0.40]	[− 0.08, 0.44]	[0.27, 0.68]	[0.20, 0.64]	[0.33, 0.71]		[0.17, 0.62]
7. Opportunity to perform	3.06	1.23	0.21	0.05	0.56^**^	0.47^**^	0.61^**^	0.42^**^	
			[− 0.07, 0.45]	[− 0.22, 0.32]	[0.35, 0.72]	[0.23, 0.66]	[0.40, 0.75]	[0.17, 0.62]	

Note: *N* = 53. Correlations for Condition 1 are presented below the diagonal; correlations for Condition 2 are presented above the diagonal. Values in square brackets indicate the 95% confidence interval for each correlation. The * symbol indicates *p* < 0.05. The ^**^ symbol indicates *p* < 0.01

**Table 3:** Correlation and reliability of study variables for Condition 3 written/open-ended and Condition 4 interactive/open-ended

	*M*	*SD*	1	2	3	4	5	6	7
*M*			1.68	29.56	5.27	5.49	3.49	4.82	4.22
*SD*			0.47	7.68	1.17	1.09	1.38	1.24	1.24
1. Gender is female	1.67	0.48		0.04	− 0.00	0.19	− 0.07	0.06	0.22
				[− 0.24, 0.31]	[− 0.28, 0.28]	[− 0.09, 0.45]	[− 0.34, 0.21]	[− 0.23, 0.33]	[− 0.07, 0.47]
2. Age	26.71	4.48	0.07		0.09	0.15	0.00	− 0.10	0.11
			[− 0.20, 0.34]		[− 0.19, 0.36]	[− 0.13, 0.41]	[− 0.28, 0.28]	[− 0.37, 0.18]	[− 0.17, 0.38]
3. Need for autonomy	4.77	1.11	0.24	− 0.07		0.20	0.35^*^	0.45^**^	0.48^**^
			[− 0.03, 0.49]	[− 0.34, 0.21]		[− 0.08, 0.45]	[0.08, 0.57]	[0.19, 0.65]	[0.24, 0.67]
4. Need for competence	5.27	1.17	− 0.06	− 0.09	0.38^**^		0.05	0.31^*^	0.41^**^
			[− 0.33, 0.22]	[− 0.36, 0.19]	[0.11, 0.59]		[− 0.23, 0.33]	[0.04, 0.54]	[0.14, 0.62]
5. Need for relatedness	3.05	1.57	− 0.16	− 0.12	0.33^*^	0.40^**^		0.50^**^	0.42^**^
			[− 0.41, 0.12]	[− 0.39, 0.16]	[0.06, 0.56]	[0.13, 0.61]		[0.25, 0.68]	[0.16, 0.62]
6. Interpersonal warmth	3.79	1.68	0.06	− 0.16	0.47^**^	0.44^**^	0.72^**^		0.46^**^
			[− 0.22, 0.33]	[− 0.41, 0.12]	[0.22, 0.66]	[0.19, 0.64]	[0.56, 0.83]		[0.21, 0.65]
7. Opportunity to perform	3.93	1.51	0.09	− 0.02	0.49^**^	0.55^**^	0.54^**^	0.61^**^	
			[− 0.19, 0.36]	[− 0.30, 0.25]	[0.24, 0.67]	[0.32, 0.72]	[0.31, 0.71]	[0.40, 0.76]	

Note: *N* = 53. Correlations for Condition 3 are presented below the diagonal; correlations for Condition 4 are presented above the diagonal. Values in square brackets indicate the 95% confidence interval for each correlation. The * symbol indicates *p* < 0.05. The ^**^ symbol indicates *p* < 0.01

### Differences in Applicant Reactions Depending on Method Factors

**Fig. 1:**
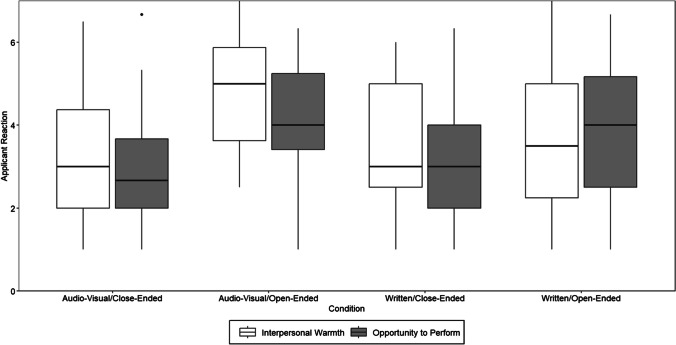
Mean applicant reaction depending on condition. Note: *N* = 208. Hinges represent the first and third quartiles; whiskers represent the 95% confidence interval; dots represent outliers. All reactions rated on a scale from 1—*completely disagree* to 7—*completely agree*

Hypothesis 1 postulated that perceptions of interpersonal warmth will be more positive for conditions with an interactive (as opposed to written) stimulus format. Mean differences in both applicant reaction dimensions are displayed in the boxplots in Fig. 1. A two-way analysis of variance (ANOVA) with stimulus format (written and interactive) and response format (close-ended and open-ended) as factors showed no significant main effect of stimulus format on perceptions of interpersonal warmth (*F*(1) = 1.29, *p* = 0.257, *η*^2^ = 0.00, 95% confidence interval (CI) [0.00, 1.00]). Therefore, Hypothesis 1 was not supported.

**Fig. 2:**
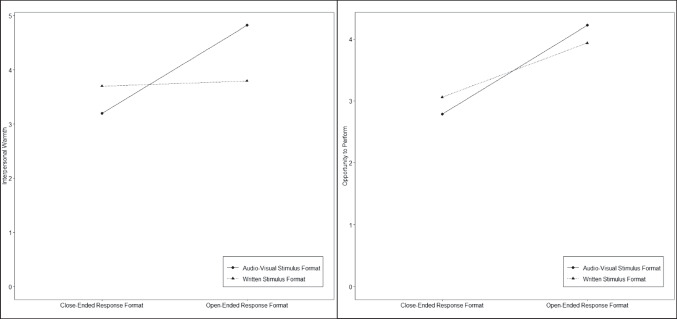
Interaction of stimulus and response format for interpersonal warmth and opportunity to perform. Note: *N* = 208. Main effect of response format on interpersonal warmth: (*F*(1) = 17.92, *p* < 0.001, *η*^2^ = 0.08, 95% CI [0.03, 1.00]). Interaction effect between stimulus and response format (*F*(1) = 14.16, *p* < 0.001, *η*^2^ = 0.06, 95% CI [0.02, 1.00]). Main effect of stimulus format on perceptions of interpersonal warmth (*F*(1) = 1.29, *p* = 0.257, *η*^2^ = 0.00, 95% CI [0.00, 1.00]) was not significant. Main effect of response format on opportunity to perform (*F*(1) = 39.98, *p* < 0.001, *η*^2^ = 0.16, 95% CI [0.09, 1.00]). Main effect for stimulus format (*F*(1) = 0.01, *p* = 0.944, *η*^2^ = 0.00, 95% CI [0.00, 1.00]), or the interaction of stimulus and response format (*F*(1) = 2.33, *p* = 0.129, *η*^2^ = 0.00, 95% CI [0.00, 1.00]) were not significant

Beyond our assumptions, the analysis revealed a non-hypothesized main effect of response format on interpersonal warmth (*F*(1) = 17.92, *p* < 0.001, *η*^2^ = 0.08, 95% CI [0.03, 1.00]) and a significant interaction effect between stimulus and response format (*F*(1) = 14.16, *p* < 0.001, *η*^2^ = 0.06, 95% CI [0.02, 1.00]). An interaction diagram is displayed in Fig. 2. Post hoc Tukey tests revealed the interactive/open-ended condition (i.e., the condition similar to a situational videoconference interview, see Table 1; *M* = 4.82, *SD* = 1.24) was rated as significantly warmer compared to all other conditions, namely the written/open-ended condition (*M* = 3.79, *SD* = 1.68, *p* = 0.003), the written/close-ended condition (*M* = 3.70, *SD* = 1.43, *p* < 0.001), and the interactive/close-ended condition (*M* = 3.19, *SD* = 1.47, *p* < 0.001). This indicates that the combination of the interactive stimulus format and open-ended response format was pivotal for perceptions of interpersonal warmth.

Hypothesis 2 postulated that participants would perceive a higher level of opportunity to perform in conditions with an open-ended (as opposed to a close-ended) response format. We tested this hypothesis with the same two-way ANOVA as for Hypothesis 1 with opportunity to perform as the dependent variable. Results revealed a significant main effect of response format on opportunity to perform (*F*(1) = 39.98, *p* < 0.001, *η*^2^ = 0.16, 95% CI [0.09, 1.00]). As can be seen in Fig. 2, this effect was in the expected direction, meaning that participants perceived conditions with an open-ended response format more positively in terms of opportunity to perform. Tukey post hoc test indicated that the videoconference/open-ended condition (*M* = 4.22, *SD* = 1.24) and the written/open-ended condition (*M* = 3.93, *SD* = 1.51) were perceived as providing significantly more opportunity to perform compared to the written/close-ended condition (*M* = 3.06, *SD* = 1.23, *p* < 0.001 and 0.005, respectively), and the videoconference/close-ended condition (*M* = 2.78, *SD* = 1.24, both *p* < 0.001). Therefore, Hypothesis 2 was supported.

Beyond Hypothesis 2, the ANOVA revealed no significant effects, meaning that there were no significant effects for stimulus format (*F*(1) = 0.01, *p* = 0.944, *η*^2^ = 0.00, 95% CI [0.00, 1.00]) or the interaction of stimulus and response format (*F*(1) = 2.33,* p* = 0.129, *η*^2^ = 0.00, 95% CI [0.00, 1.00]) on opportunity to perform.

### Differences in Need Satisfaction Depending on Method Factors

**Fig. 3:**
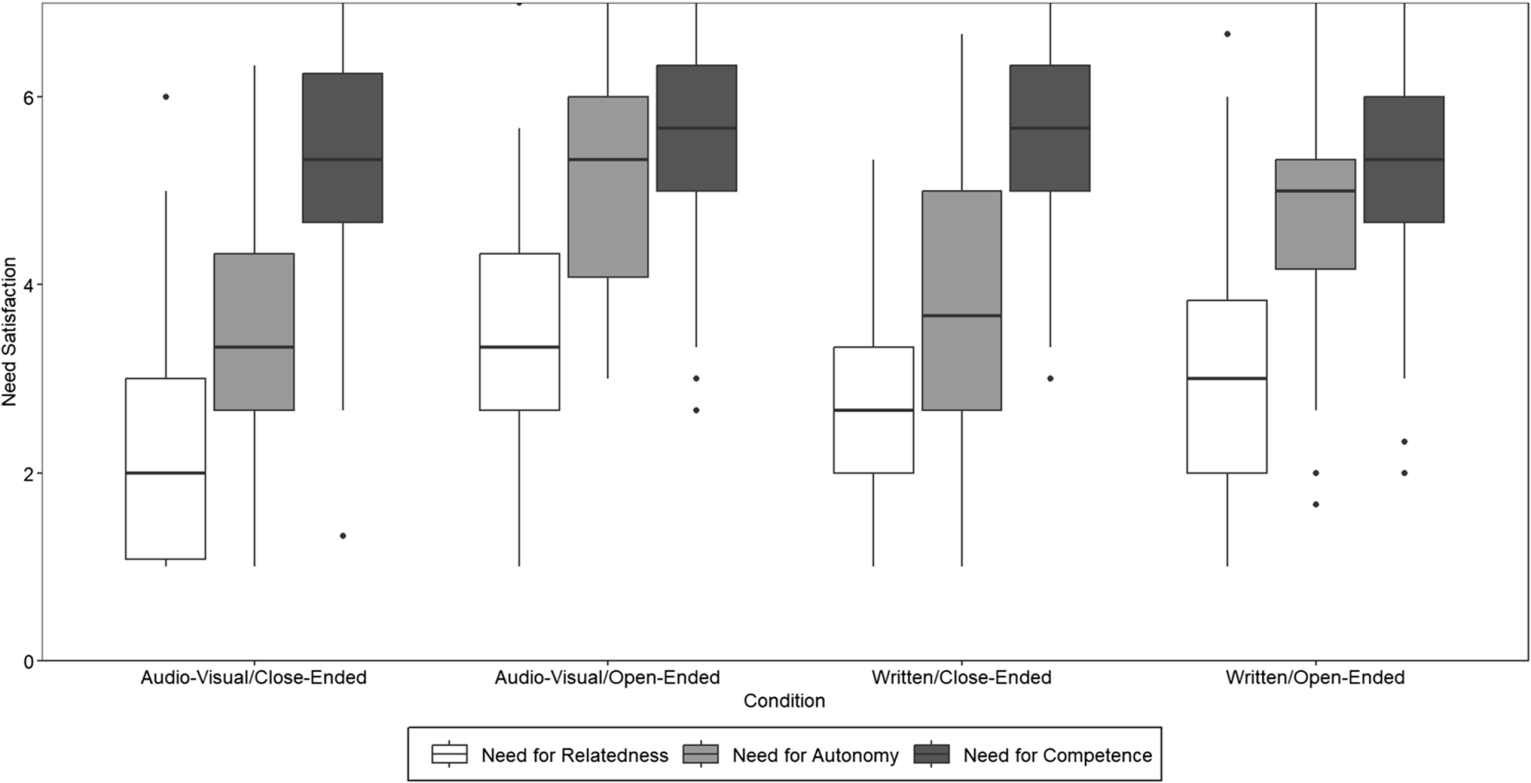
Mean satisfaction of need for autonomy, competence, and relatedness depending on condition. Note: *N* = 208. Hinges represent the first and third quartiles; whiskers represent the 95% confidence interval; dots represent outliers. All reactions rated on a scale from 1—*completely disagree* to 7—*completely agree*

Hypotheses 3 and 4 postulated differences in need satisfaction across method factors. Mean differences for need satisfaction across conditions are displayed in the boxplots in Fig. 3. Hypothesis 3 postulated that an interactive stimulus format will better satisfy need for relatedness compared to a written stimulus format. We analyzed the differences between these method factors using planned contrasts. Results are displayed in Table 4. Planned contrasts revealed no significant differences between conditions that differed only in stimulus format (*t* = 1.38, *p* = 0.170, 95% CI [− 0.27, 1.49]). The descriptive pattern also contradicted the assumed ranking: although the interactive/open-ended condition best satisfied need for relatedness (*M* = 3.49, *SD* = 1.38), the interactive/close-ended condition least satisfied need for relatedness (*M* = 2.36, *SD* = 1.17). Thus, Hypothesis 3 was not supported.

**Table 4:** Results of contrast tests

Outcome	Contrast					*t*	*p*	*95% CI*
	Effect of	Condition						
		1	2	3	4			
Need for relatedness	Stimulus format	0	1	0	1	1.38	0.170	− 0.27, 1.49
Need for autonomy	Response format	0	0	1	1	5.38^**^	< 0.001	1.53, 3.30
Need for competence	Response format	0	0	1	1	− 0.99	0.391	− 1.18, 0.38

Note: *N* = 208; contrasts comparing conditions with the same response format were 0011; contrasts comparing conditions with the same stimulus format were 0101. Condition 1 = close-ended/written, Condition 2 = close-ended/interactive, Condition 3 = open-ended/written, Condition 4 = open-ended/interactive. *p < 0.05, **p < 0.01

Hypotheses 4 postulated that conditions with open-ended response format will better satisfy (a) need for autonomy and (b) need for competence. For need for autonomy, planned contrasts in Table 4 showed significant differences between methods with the same stimulus format and different response format, namely between the written/close-ended (*M* = 3.81; *SD* = 1.56) and the written/open-ended condition (*M* = 4.77; *SD* = 1.11; *p* = 0.002, 95% CI [0.29, 1.63]), and between the interactive/close-ended (*M* = 3.41, SD = 1.39) and the interactive/open-ended condition (*M* = 5.27, *SD* = 1.17;* p* < 0.001, 95% CI [1.18, 2.53]). In both cases, need satisfaction was higher in conditions with open-ended response formats, supporting Hypothesis 4a.

For need for competence, planned contrasts revealed no significant differences between conditions with open-ended as opposed to close-ended response formats (*t* = − 0.99, *p* = 0.391, 95% CI [− 1.18, 0.38]). Accordingly, Hypothesis 4b was rejected.

### Need Satisfaction as Mediator Between Method Factors and Applicant Reactions

Hypotheses 5 and 6 postulated that satisfaction of needs will mediate the relationship between method factors and applicant reactions. We analyzed these hypotheses using mediation analyses with Hayes PROCESS model 4 (Hayes, 2017). Indirect effects were estimated and standardized using bootstrapping with 10,000 bootstraps. At the outset, similar to prior research (Borman et al., 2023), we would like to point out that need satisfaction and applicant reactions had to be measured at the same time point in the study (i.e., after the completion of the selection instrument) to capture direct reactions, which warrants caution for drawing causal conclusions.

**Fig. 4:**
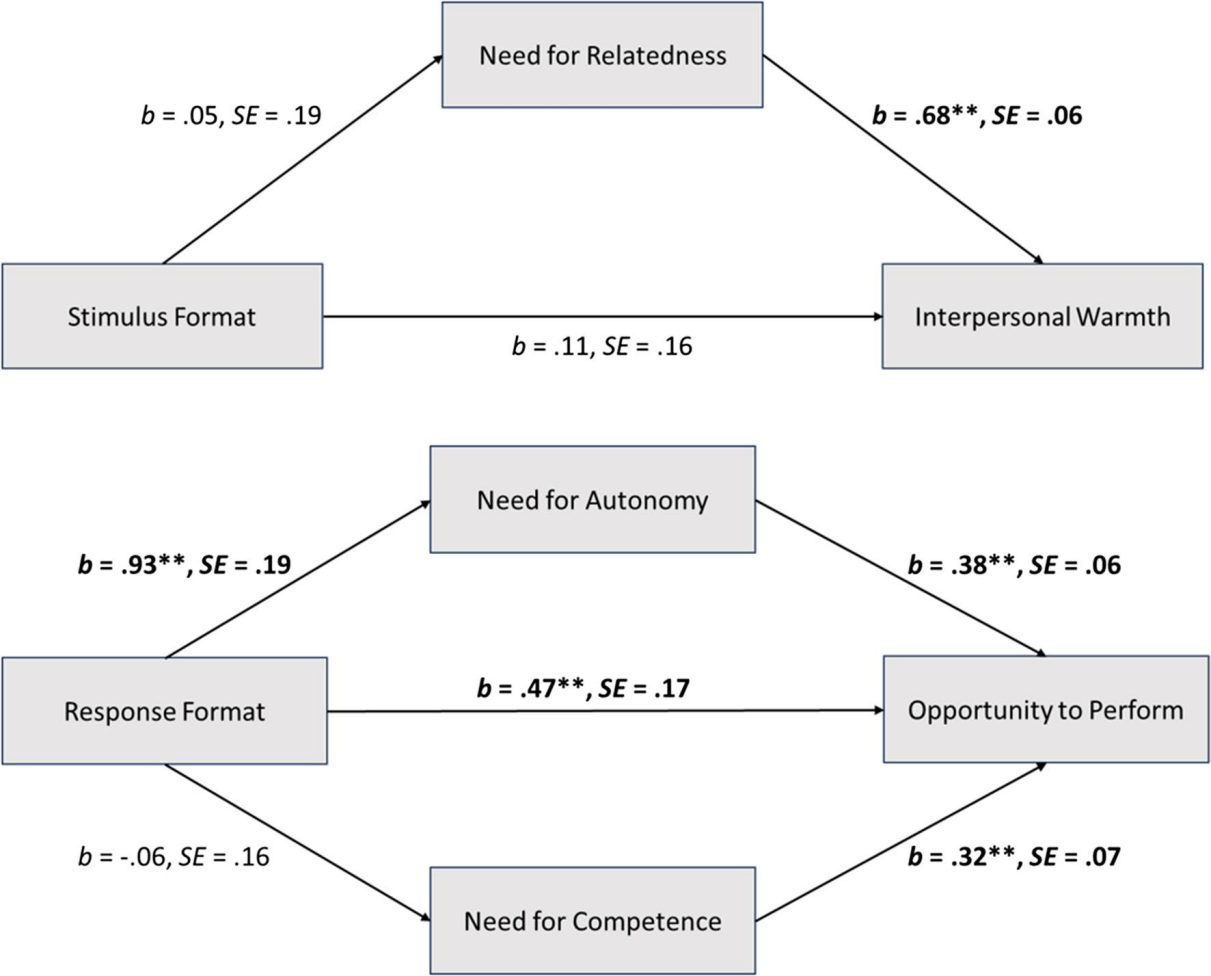
Mediation model for Hypotheses 4–6. Note: *N* = 208. Significant paths are printed in bold. All effect sizes are standardized. For the model with interpersonal warmth, the standardized indirect effect was *b* = 0.04, *SE* = 0.09 and the total effect was *b* = 0.15, *SE* = 0.22.For the model with opportunity to perform, the total indirect effect of both mediators was *b* = 0.43, *SE* = 0.13, the standardized indirect effect via need for autonomy was *b* = 0.35, *SE* = 0.07, the standardized indirect effect via need for competence was *b* = − 0.02, *SE* = 0.07 and the total effect *b* = 0.80, *SE* = 0.18. The * symbol indicates *p* < 0.050; the ** symbol indicates *p* < 0.001

Hypothesis 5 assumed a mediation effect of need for relatedness in the relationship between stimulus format and perceived interpersonal warmth. All mediation results are reported in Table 5. The mediation models are displayed in Fig. 4. The indirect effect via need for relatedness was not significant, and, thus, Hypothesis 5 was rejected.

**Table 5:** Test of direct and indirect effects of mediation analyses

Interpersonal warmth										
	*b*			*SE*		*t*		*p*	*R*^2^	
Direct effects										
Stimulus format → need for relatedness	0.05			0.19		0.039		0.700		0.00
Stimulus format → interpersonal warmth	0.11			0.16		1.09		0.278		0.47^**^
Need for relatedness → interpersonal warmth	0.68			0.06		13.30		< 0.001		
Total effect	0.15			0.022		1.06		0.290		0.07
Indirect effect				Boot *SE*		Boot *LLCI*		Boot *ULCI*		
Stimulus format → need for relatedness → interpersonal warmth	0.04			0.09		− 0.14		0.22		
Opportunity to perform										
	*b*			*SE*		*t*		*p*		*R*^2^
Direct effects										
Response format → need for autonomy	0.93			0.19		7.58		< 0.001		0.22^**^
Response format → need for competence	− 0.06			0.16		− 0.43		0.671		0.00
Response format → opportunity to perform	0.47			0.17		3.92		< 0.001		0.45^**^
Need for autonomy → opportunity to perform	0.38			0.06		6.03		< 0.001		
Need for competence → opportunity to perform	0.32			0.07		5.66		< 0.001		
Total effect	0.80			0.18		6.32		< 0.001		0.40^**^
Indirect effect				Boot *SE*		Boot *LLCI*		Boot *ULCI*		
Response format → need for autonomy and need for competence → opportunity to perform	0.34			0.09		0.16		0.52		
Response format → need for autonomy → opportunity to perform	0.35			0.07		0.23		0.50		
Response format → need for competence → opportunity to perform	− 0.02			0.07		− 0.11		0.06		

Note: *N* = 208. Standardized estimates reported. Indirect effects were estimated using 10,000 bootstraps. *LLCI* lower limit confidence interval, *ULCI* upper limit confidence interval. *p < 0.05., **p < 0.01

Hypothesis 6 assumed a mediation effect of need for autonomy (Hypothesis 6a) and need for competence (Hypothesis 6b) on the relationship between response format and perceived opportunity to perform. We performed a parallel mediation analysis with both mediator variables. The results of the mediation analyses are reported in Table 5 and the mediation model is displayed in Fig. 4. The total indirect effect of both mediators was significant, indicating that one or both needs served as a mediator.

The indirect effect mediated by need for autonomy, as assumed by Hypothesis 6a, was significant. Response format significantly predicted satisfaction of need for autonomy, which in turn predicted opportunity to perform. Thus, results confirmed that applicants perceived an open-ended response format as giving them a better opportunity to perform and that this effect was mediated by satisfaction of need for autonomy, which supported Hypothesis 6a.

The indirect effect mediated by need for competence, as assumed by Hypothesis 6b, was not significant, as indicated by the confidence interval including zero. Accordingly, Hypothesis 6b was not supported.

### Full Model

**Fig. 5:**
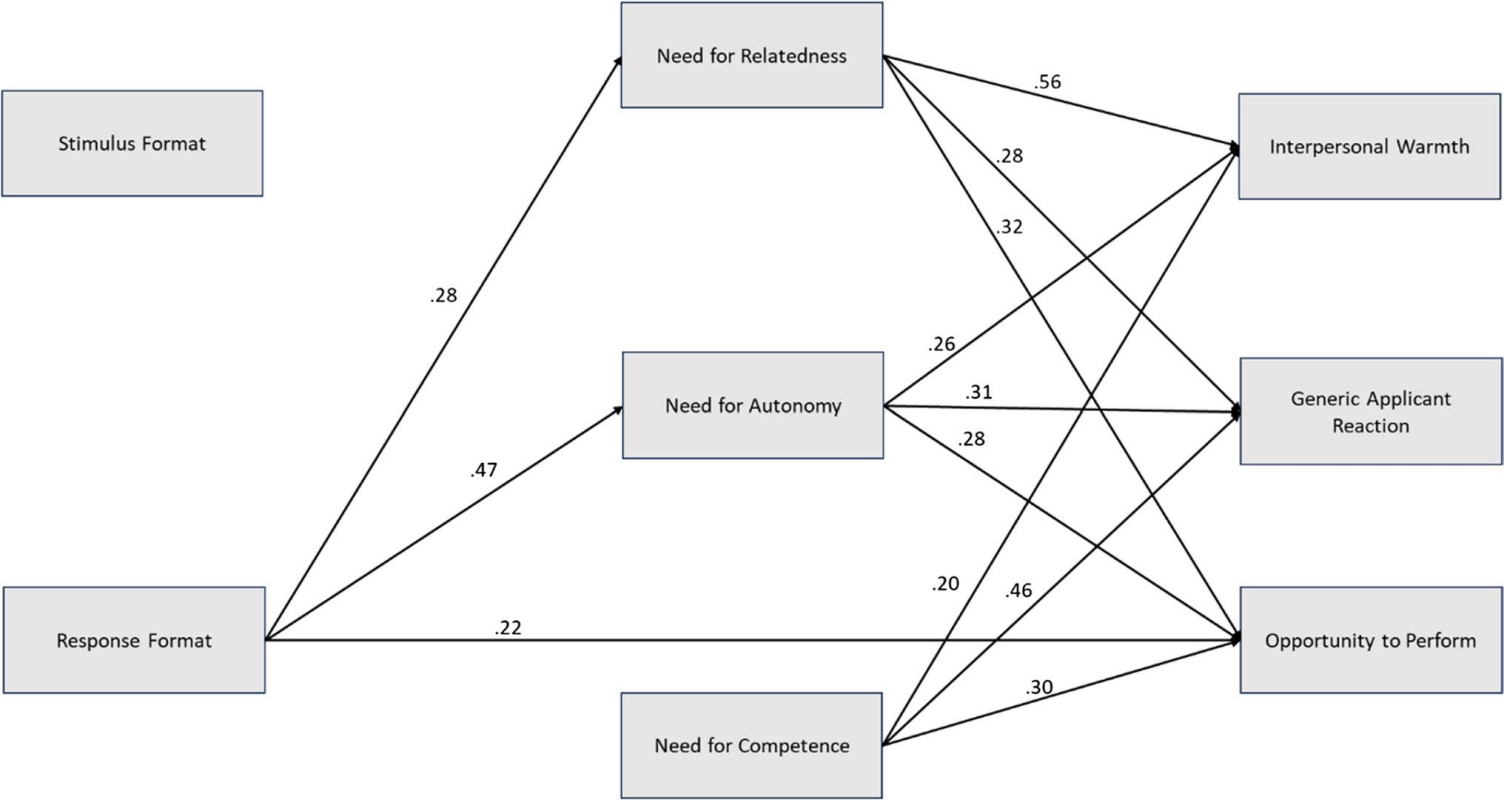
Results of the full model combining all hypotheses. Note: *N* = 208. For better visibility, only significant paths are displayed. The item used for measuring the generic applicant reaction was “Overall, how did you like the instrument?”

**Fig. 6:**
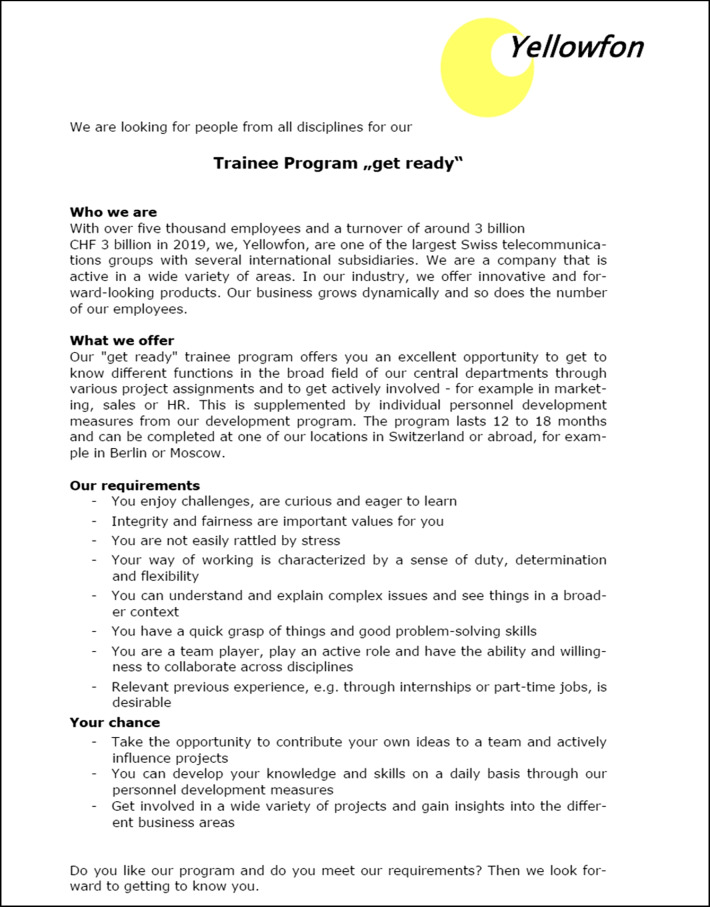
Job description sent to participants

As additional, exploratory analyses, we conducted a path model in which we tested effects of stimulus and response format on satisfaction of all three needs and both applicant reaction dimensions simultaneously. In addition, we included a generic applicant reaction item. The model and results are presented in Fig. 5. The results replicate the above-presented findings concerning effects on interpersonal warmth and opportunity to perform. Stimulus format did not significantly affect any of the three needs, nor the applicant reaction outcomes. Response format had a significant effect on satisfaction of need for autonomy and need for relatedness and a direct effect on opportunity to perform. The results further indicated that need satisfaction affected generic applicant reaction, but there was no direct effect of stimulus or response format.

## Discussion

Although the use of well-accepted personnel selection instruments is important for attracting talented applicants (Uggerslev et al., 2012), there is a lack of comprehensive scientific evidence on the underlying processes of applicant reactions, in particular concerning method factors that characterize selection instruments. Prior applicant reaction research has focused on applicants’ general preference for holistic selection instruments and found that interviews and work samples serve as the most preferred instruments, whereas psychometric tests are rated as less favorable. The present study qualifies prior knowledge by scrutinizing the psychological processes underlying reactions to specific method factors. Specifically, this study examined how applicant reactions are mediated by applicants’ need satisfaction as a potential explaining mechanism. This study is the first to isolate the effects of two important method factors (stimulus and response format) in an experimental design with participants rating their reactions after completing the selection instrument in question. As such, the results of this study go beyond and explain former findings on the favorability of interviews (e.g., Anderson et al., 2010) and illustrate that the open-ended response format, rather than the stimulus format, is responsible for these reactions. Specifically, the open-ended response format led to a greater opportunity to perform and interpersonal warmth both directly but also indirectly through satisfaction of need for autonomy. It thus adds to the understanding of previous work and illustrates the value of the nuanced approach taken in this study.

### Key Findings and Theoretical Implications

The first key finding of this study is that the response format of a selection instrument appears to be more relevant for applicant reactions than its stimulus format. In the open-ended response format, as opposed to the close-ended response format, applicants perceived a higher opportunity to perform, and non-hypothesized, more interpersonal warmth in the selection instrument. The stimulus format, on the contrary, showed no significant effect on either outcome. Conceptually, this may suggest that method factors affecting applicants’ responses (i.e., the active part of completing a selection instrument) contribute more to their reactions to this instrument compared to a method factor that entails how something is presented to them (i.e., a more distal factor). This might also explain portions of the favorability of the interview (Anderson et al., 2010). Based on the present findings from a research design that allowed for isolating the impact of method factors, while keeping the measured construct constant, it appears plausible that job interviews—at least in parts—are perceived so favorably because they are the selection instruments that are most commonly characterized by an open-ended response format. On a theoretical level, the findings also strengthen past conjectures that the method used exerts an impact on applicant reactions that is independent of the construct measured (Hausknecht, 2013) and provide an empirical underpinning for the importance of avoiding content-method confusion (Arthur & Villado, 2008) in applicant reaction research.

Looking more closely at the unexpected effect of response format on interpersonal warmth, this relationship could be explained post hoc as a signal of the organization’s interest in the applicant. Specifically, by allowing the applicants to explain themselves, the open-ended response format may have signaled to the applicants that the organization was interested in their personal explanations to each question. Applicants may perceive it as a sign of appreciation if they assume that the organization takes extra time to review their self-phrased responses inherent to an open-ended response format. Yet, because we did not hypothesize this effect a priori, future research may wish to replicate and test this interpretation.

Second, the combination of stimulus and response format also mattered, especially for interpersonal warmth perceptions. Specifically, we found a non-hypothesized interaction effect between stimulus and response format: applicants did not perceive a method as more personal only because the stimulus was presented by a person in an interactive stimulus format, but they did when this interactive stimulus was combined with an open-ended response format. The higher favorability of the interview-like combination may suggest that stimulus and response formats should be combined in a familiar way. Unusual combinations of different method factors (such as an interactive stimulus format and a close-ended response format) may be more negatively perceived because they feel unfamiliar or artificial. Accordingly, perceptions of how widely used an instrument is are considered another dimension of applicant reactions (Gilliland & Steiner, 2012), and unfamiliarity of selection instruments has been associated with a feeling of “creepiness” (Langer et al., 2017). In this study, the videoconference stimulus format with open-ended responses may have been closer to a familiar conversation setting, even though the interview was fully standardized (i.e., also not allowing for fully natural conversation). In contrast, the combination of an interactive stimulus format with a close-ended response format, which resulted from our systematic experimental manipulation, meant that applicants saw someone in a videoconference setting but could not formulate whole sentences as in a typical conversation-like setting. Overall, these exploratory findings also highlight that the combination of method factors needs to be considered.

As a third key finding, satisfaction of need for autonomy mediated the effect of response format on applicant reactions, whereas mediations with needs for autonomy and competence did not reach significance. Conceptually, this suggests that allowing applicants to formulate their answer is of particular relevance for making them feel in control of the situation. For SDT, the findings add to the first efforts placing applicant reactions to the list of work-related outcomes affected by satisfaction of needs. It also extends recent research on SDT in applicant reactions (Borman et al., 2023; Buil et al., 2020), as the first study to explicitly integrate a method factor approach with SDT, and to uncover direct links between specific method factors, specific needs, and specific applicant reaction dimensions. Thereby, the findings call for an extension of theoretical models of applicant reactions findings two ways: first, the results of this study confirm the pivotal role of need for autonomy, which is considered by some as the most central need within SDT theory (Yu et al., 2018), extending it to the context of applicant reactions. Therefore, it suggests introducing need for autonomy, as a mediator in theoretical models between antecedents and applicant reactions (e.g., Hausknecht, 2013). Second, in light of evidence on the different paths between method factors and needs, it suggests to incorporate method factors in theorizing on applicant reactions. On the basis of these two extensions, we hope to encourage subsequent efforts to explore how construct or method changes may affect the satisfaction of other needs.

Fourth, and unexpectedly, we found that the satisfaction of all three needs (i.e., needs for relatedness, autonomy, and competence) had significant effects on both applicant reaction outcomes examined (interpersonal warmth and opportunity to perform). There are different explanations for this finding. On the one hand, it seems plausible that the satisfaction of all needs is relevant to applicant reactions because all three needs are relevant for human thriving and motivation in a given situation (Deci & Ryan, 2000, 2010; Van den Broeck et al., 2016). On the other hand, the question arises as to the extent to which the satisfaction of the three needs is independent of each other, which has been subject to discussion in the past (Van den Broeck et al., 2016). Despite being conceptually distinct, the three needs are often examined in a sum score and show comparably strong relationships to third variables (Deci et al., 2001; Gagné, 2003). The finding that all needs are significantly related to all applicant reactions (instead of only those we conceptually expected), raises the question of whether the satisfaction of one isolated need can be targeted, or whether the satisfaction of one need affects the satisfaction of other needs.

### Practical Implication

For practitioners, results from this study first and foremost underscore the importance of making conscious decisions when it comes to the measurement of predictor constructs. Choosing a favorable way to measure constructs enables organizations to assess even less favorable constructs without compromising on acceptance in their selection process. Specifically, based on these findings, a favorable method is characterized by an opportunity for applicants to explain themselves. After ensuring that the choice of the method does not negatively affect criterion-related validity, organizations could achieve improvements by choosing instruments that allow the applicant to verbalize their responses to a question. Even in the absence of any interaction, such adjustments can lead to improved reactions, although an interview-like instrument that involves both interactions and the opportunity to explain oneself achieved the best results.

The role of need satisfaction offers more in-depth insights into applicant reactions that potentially extend beyond response formats. Results imply that giving applicants the freedom to make their own decisions within a selection instrument can be a setscrew for achieving favorable perceptions. Organizations can offer some autonomy to applicants by allowing them to respond to instruments in their own words (i.e., an open response format). Yet, they might achieve similar effect with other instruments that facilitate a great scope of action, such as unstandardized interviews or free introductory pitches from the applicants. Beyond that, instruments that feel personal and allow applicants to demonstrate their strengths can further improve reactions.

An important restriction in this context is that criterion-related validity should not be sacrificed for enhanced applicant reactions. Conceivably, instruments that allow greater latitude may come at the expense of standardization and thereby, validity (Sackett et al., 2022). At the same time, the condition that received the most favorable applicant reactions (i.e., interactive stimulus format and open-ended response format) and also allowed the greatest latitude,was similar to a typical structured interview, which has shown to be the most criterion-valid selection instrument according to a recent meta-analysis (Sackett et al., 2022). Nonetheless, organizations must weigh potential trade-off between the validity and favorability of selection instruments and ensure a valid measurement and sufficient predictive power before using or adjusting selection instruments.

For some organizations, adjusting method factors may be less feasible, and for these, adding elements that facilitate autonomous actions can be a potential alternative. Alternative strategies for compensating for the frustration of need for autonomy may bring particular benefits for organizations that obtain ready-to-use tests from external providers that are non-adjustable. For example, similar to the variations tested by Borman et al. (2023) for forced-choice personality inventories, organizations may try to add follow-up discussions to standardized tests such that applicants get the chance to elucidate their responses. By using such follow-up elements, practitioners may also be able to harness the advantage of both standardized measurements and positive applicant reactions. A greater use for our findings therefore lies in the design of selection instruments, in which favorable method factors can be considered early on and used as a basis for developing valid selection instruments.

This study shows that open-ended response formats are perceived positively, and this raises the question of how to best rate such responses effectively and efficiently (i.e., how to translate them into quantitative ratings). A traditional approach is to rate the responses using predefined examples for high and low expressions, also known as behaviorally anchored rating scales (BARS; Lubbe & Nitsche, 2019). Responses that are rated with BARS are more accurate and show a higher relationship with job performance (Taylor & Small, 2002). Because the ratings require additional effort, open-ended responses can be a sacrifice in efficiency. Yet, in light of the rising functionality of artificial intelligence for scoring open-ended responses and respective resources for organizations on how to use them (e.g., Guo et al., 2024; Hickman et al., 2022), newer technology can help to alleviate such sacrifices. As such, adjusting response format alone (as compared to implementing a full one-to-one interaction) can function as a time-flexible alternative to enhance even perceptions of interpersonal warmth.

### Limitations

This study is not without limitations. First, we used a simulated selection procedure that allowed to examine detailed effects in a standardized setting to exclude interfering influences as much as possible. Although we took care to mimic personnel selection settings as far as possible (e.g., providing an incentive to perform, interviewers wearing professional clothing) and participants indicated that they found that the offered position was interesting and that they acted as if they were in an actual selection situation, we would like to raise that reactions might still differ to some degree from real applicants (Anderson et al., 2010; Arvey et al., 1990).

Second, because all conditions of our experiment started with a greeting in the videoconference setting, participants in the written conditions may have felt that they also had an experience that allowed for contact with someone from the organization. Despite being forwarded to the written instrument afterward, this short introduction may have been a personal experience that affected results compared to a written instrument that is conducted without any first interactions. Further, because of this greeting, participants in the written conditions had to switch from videoconference to the written instrument. This switch in the setting (i.e., the step in the process where participants technically switched from one medium to the other) may have exerted an effect on participants’ perceptions in the written condition that was not present in the interactive condition. Yet, even if the greeting or the change of medium affected our results, this effect was not strong enough to influence the overall pattern of results.

Third, we deliberately focused on specific applicant reaction dimensions (namely, opportunity to perform and interpersonal warmth), which allowed us to map the manipulations of method factors to relevant outcomes. As such, our findings do not capture the broader spectrum of applicant reactions that are also critical in selection contexts. For example, examining perceived scientific evidence or face validity could offer additional insights for a holistic evaluation of selection methods (Anderson et al., 2010).

### Future Research

Building on this study, future research could focus on other outcomes affected by method factor changes and need satisfaction. The present research took a first step in showing how single method factors satisfy basic needs, subsequently influencing selection outcomes. Future research may want to delve deeper into how stimulus and response format affects the personality measurement of selection instruments concerning the quantity and quality of information (e.g., by considering interactionist theories such as trait activation theory; Tett and Guterman, 2000). Participants can share more information in open-ended response formats and also in an interactive stimulus format, affecting the potential number and quality of trait-relevant cues available to the interviewer. Need satisfaction may play a role for information richness, too: for example, the satisfaction of need for relatedness may make applicants feel more comfortable sharing information within a selection instrument.

To enhance the understanding of method factors for applicant reactions, future research could also look more closely at the circumstances under which expected effects occur in practice. Applicant reactions to an instrument are often studied in isolation (Ryan & Huth, 2008), but can be affected by the context in which they are used, such as the greeting or feedback, other instruments in the process (Rosse et al., 1994), and the stage at which they are used (Zibarras & Patterson, 2015). The same is true for need satisfaction: for example, applicants may be content if a personal greeting or other selection instruments used within the process meet their need for relatedness, in which case they may accept a less personal instrument at earlier or later stages of the process. Future research is needed to study the sequence of instruments and process factors such as rapport building.

Taking a broader view, this study is exemplary of an approach that could inspire future research to extract and examine the effects of other method factors or other content measured and their interaction on need satisfaction. Although an open-ended response format was found to be particularly favorable in this study, the effects of other method factors may complement or even outweigh the effect of the open-ended response format on applicant reactions. For example, contextualization (i.e., how much the instrument content is tailored to the relevant context, such as work) is one factor that was held constant across instruments and has shown small effects on personality measurement in previous research (Fisher et al., 2017; Holtz et al., 2005). Given its relevance for the overall favorability of the selection process (e.g., Zibarras & Patterson, 2015), it is worth examining the effects of contextualization in combination with other method factors (e.g., an open-ended response format, that allows applicants to tell a success-relevant work story) or for other constructs. Research of such design could eventually identify which combinations of method factors and content work particularly well.

### Conclusion

Understanding the impact that need satisfaction has for applicant reactions gives insights into why reactions occur and how they can be enhanced. Based on the extracted effects of stimulus and response formats, the insights of this study can provide important information for the choice and design of selection instruments. Specifically, this study showed that need for autonomy satisfaction enhances perceptions of opportunity to perform and that using methods with an open-ended response format is effective for satisfying applicants’ need for autonomy. Further, perceptions of interpersonal warmth are increased by using an interactive stimulus format in combination with an open-ended response format. We can thus conclude from our findings that selection instruments benefit from providing applicants with autonomy to give them a pleasurable experience, leading to a better perceived opportunity to perform.

## Appendix

See Tables. 6 and 7, Fig. 6

**Table 6:** Sample item for the personality question used for the experimental conditions

Item personality inventory	Situation description experimental condition with response option
I am always prepared. (conscientiousness)	Your organization has developed a new product. You are given the task of promoting the product to different target groups. To do this, you have to make the same presentation to different professional and personal groups who each have different interests in relation to the product (e.g., representatives, advertising managers, internal and external customers). At the same time, you also have the task of preparing reports on the product and have to spend a lot of time on this. How would you personally experience this situation and how would you behave in this situation with regard to preparing for the presentations? 1. As I don’t want to get stressed by the deadlines of my other tasks, I don’t prepare for the individual presentations. As I have to give them several times anyway, the preparation comes naturally 2. As soon as I have the presentation more or less in my head and know that I have mastered the content, I no longer look at it before the deadlines because I don’t want to neglect my other tasks 3. I briefly look at the presentation again before each appointment to prepare. On the way to the respective presentation date, I think about how I can make references to the target group. However, I don’t make any major changes in the presentation as I have to concentrate on the other tasks 4. Although I also have to concentrate on the other tasks, I prepare the presentation again before each appointment and adapt it slightly to the interests of the target group 5. I take time before each appointment to revise the presentation with regard to the relevant target group and make references to the respective needs of the target group. If possible, I try not to neglect the other tasks
I feel comfortable around people. (extraversion)	You work in a large building in which various teams from your organization are based. Every year, a summer party is organized to which all the teams are invited. All companies close in the afternoon for this event. The party is intended to promote interdisciplinary exchange; participation is voluntary but encouraged. However, you have some time-critical administrative work to do, for which you could make good use of the peace and quiet at your workplace. How would you personally experience the situation with regard to participation in the festival and how would you behave? 1. I don’t enjoy such events very much in comparison and would only have limited pleasure in having to talk to other people or new people. I will probably use the time to do my other tasks 2. I don’t necessarily look forward to forced small talk. Nevertheless, I will go to the summer party for a few minutes, even if I would rather get on with my other tasks 3. I think the summer party is a good idea and will go. If I realize that it's not useful for me and I can’t make any new contacts, I might leave early to take care of other things 4. I think I will enjoy talking to my neighbors and would like to take the opportunity to network, therefore, I will take part in the party. I'm happy to put my administrative tasks on hold 5. I’m really looking forward to the festival and the exchange with the other teams and hope to make some new contacts there. As I find such events important and enjoy them, I would always prefer them to other tasks

Note: Response options in order of trait expression with 5 = highest. Response options were only shown in the conditions with close-ended response format

**Table 7:** Items for need satisfaction

Need for relatedness
I don't really feel connected to the people in this organization while completing this selection instrument
While completing this selection instrument, I feel part of a group
I feel a sense of connection while completing this selection instrument
Need for autonomy
This selection instrument allows me room for maneuver
I can decide for myself how I complete this selection instrument
The possibility to make your own decisions is given in this selection instrument
Need for competence
I feel competent and with the necessary knowledge while completing this selection instrument
I feel capable of performing successfully in this selection instrument
My skills fit well with the requirements of the selection instrument

Note: All items rated on a scale from* 1—completely disagre*e to *7—completely agree*
